# IL-22 regulates endometrial regeneration by enhancing tight junctions and orchestrating extracellular matrix

**DOI:** 10.3389/fimmu.2022.955576

**Published:** 2022-08-25

**Authors:** Umida Ganieva, Sylvia Schneiderman, Pengli Bu, Kenneth Beaman, Svetlana Dambaeva

**Affiliations:** ^1^ Center for Cancer Cell Biology, Immunology, and Infection, Rosalind Franklin University of Medicine and Science, North Chicago, IL, United States; ^2^ Clinical Immunology Laboratory, Rosalind Franklin University of Medicine and Science, North Chicago, IL, United States; ^3^ Department of Pharmaceutical Sciences, College of Pharmacy, Rosalind Franklin University of Medicine and Science, North Chicago, IL, United States

**Keywords:** claudin-2, claudin-10, IL-22, IL-22^-/-^ mice, lipopolysaccharide, mucin-1, tight junctions, uterine regeneration

## Abstract

The uterine endometrium uniquely regenerates after menses, postpartum, or after breaks in the uterine layer integrity throughout women’s lives. Direct cell–cell contacts ensured by tight and adherens junctions play an important role in endometrial integrity. Any changes in these junctions can alter the endometrial permeability of the uterus and have an impact on the regeneration of uterine layers. Interleukin 22 (IL-22) is a cytokine that is recognized for its role in epithelial regeneration. Moreover, it is crucial in controlling the inflammatory response in mucosal tissues. Here, we studied the role of IL-22 in endometrial recovery after inflammation-triggered abortion. Fecundity of mice was studied in consecutive matings of the same animals after lipopolysaccharide (LPS) (10 µg per mouse)-triggered abortion. The fecundity rate after the second mating was substantially different between IL-22 knockout (IL-22^−/−^) (9.1%) and wild-type (WT) (71.4%) mice (p < 0.05), while there was no difference between the groups in the initial mating, suggesting that IL-22 deficiency might be associated with secondary infertility. A considerable difference was observed between IL-22^−/−^ and WT mice in the uterine clearance following LPS-triggered abortion. Gross examination of the uteri of IL-22^−/−^ mice revealed non-viable fetuses retained inside the horns (delayed clearance). In contrast, all WT mice had completed abortion with total clearance after LPS exposure. We also discovered that IL-22 deficiency is associated with a decreased expression of tight junctions (claudin-2 and claudin-10) and cell surface pathogen protectors (mucin-1). Moreover, IL-22 has a role in the remodeling of the uterine tissue in the inflammatory environment by regulating epithelial–mesenchymal transition markers called E- and N-cadherin. Therefore, IL-22 contributes to the proper regeneration of endometrial layers after inflammation-triggered abortion. Thus, it might have a practical significance to be utilized as a treatment option postpartum (enhanced regeneration function) and in secondary infertility caused by inflammation (enhanced barrier/protector function).

## Introduction

The endometrium is a unique tissue that undergoes monthly cycles of proliferation, differentiation, breakdown, shedding, and repair after menstruation, following childbirth, or premature pregnancy loss (1). Epithelial progenitor cells and perivascular mesenchymal stem cells mediate regeneration of the endometrium (2) that undergoes mesenchymal–epithelial transition (MET) to restore endometrial homeostasis (3). Endometrial damage due to infections and decreased regenerative ability is considered among the important factors contributing to the pathogenesis of female fertility issues like recurrent pregnancy loss (RPL) and recurrent implantation failure (RIF) (4). However, the ability of the endometrium to regenerate has yet to be confirmed in terms of mechanism and regulatory factors.

The extraordinary plasticity of the endometrium is accompanied by cell–cell contact changes of tight and adherens junctions, to meet the respective requirements of the transforming uterine layers (5). Cell–cell contacts assist in establishing pregnancy (by transforming the endometrium into a receptive state), regenerating postpartum (polarity issues), and changing throughout the estrous cycle (morphological tightness). The combination and ratio, as well as the amount and localization of tight and adherens junctions, change through the cycle and ultimately determine the permeability characteristics of the endometrium. These are key factors in embryo implantation and receptivity (4). Claudins are located in the apicolateral membranes of epithelial and endothelial cells and contribute to strand formation and the fence barrier function of tight junctions (6). The claudin family includes functional proteins that are important components of tight junctions. Being a continuous intercellular barrier between epithelial cells, claudins separate tissue spaces to enhance or reduce the permeability of solutes across the epithelium selectively. Therefore, claudins can be “pore forming” or “sealing” (7). Several members of the claudin family, including claudin-3, claudin-7, and claudin-10, have different expression patterns in the endometrium during various phases of the menstrual cycle and during pregnancy, during decidualization and trophoblast invasion (8). However, another report states that pregnancy can be established successfully in the absence of claudins (9). Therefore, “sealing” and “pore-forming” claudins (7) might have far more complex functionality, especially in the response to various internal and external stimuli.

Upregulation of claudin-2 is observed in intestinal mucosa in response to enteric infection. Claudin-2 promotes para-cellular sodium and water efflux, resulting in diarrhea, which facilitates pathogen clearance. Claudin-2 transcriptional regulation analyses showed a significant role of interleukin 22 (IL-22) (10). IL-22 is usually produced at the site of inflammation by Th22, Th17, or innate lymphoid cell type 3 (11–15). It is a cytokine with double activity: 1) activates the immune system and 2) contributes to the enhanced production of extracellular matrix components. These are involved in tissue regeneration by promoting the proliferation and survival of epithelial cells (16). When IL-22 was first discovered, it was named IL-10-related T cell-derived inducible factor (IL-TIF) (17). IL-23, IL-22 binding protein, IL-1β, Notch signaling pathway, and aryl hydrocarbon receptor are the main molecules that induce or reduce the expression of IL-22 (18). In addition to the intestine, IL-22 is involved in the pathophysiology of connective tissue diseases, autoimmune disorders, and carcinogenesis (16, 19, 20). The receptor for IL-22 is primarily detected on epithelial and stromal cells. Signaling through IL-22 receptor involves the activation of Janus kinase 2, tyrosine kinase 2, with the phosphorylation of STAT-1, STAT-3, and STAT-5 (18).

In our previous work, we identified the importance of IL-22 in controlling the uterine reaction to bacterial endotoxin exposure in late pregnancy (21). IL-22 knockout mice were highly susceptible to lipopolysaccharide (LPS) treatment. We also found that recombinant IL-22 treatment significantly reduces the risk of LPS-induced abortion or intra-uterine death by preventing LPS-induced activation of the extrinsic pathway of apoptosis at the feto-maternal interface at late gestation (21). In our current study, we hypothesized that IL-22 is involved in the regeneration of uterine mucosal tissue after inflammation-triggered abortion. We found that IL-22 functions in close contact with cell surface pathogen protector mucin-1, tight and adherens junctions, and mesenchymal–epithelial transition markers to repair the endometrium following LPS treatment. The inflammatory changes after LPS-induced abortions are accompanied by altered functions of tight junctions like increased permeability and “sealing” the extracellular space. IL-22 is important for the regeneration of endometrial layers during normal cycling. IL-22 is key in order to prevent secondary infertility caused by endotoxin-induced inflammation after inflammatory-induced abortions.

## Material and methods

### Animals

C57BL/6J (wild type (WT)) and IL-22 knockout (IL-22^−/−^) (mixed background of 129S5/SvEvBrd and C57BL/6; Genentech, CA, USA) mice of 7–14 weeks of age were used in this study. The animals were maintained under a 12-h light–dark cycle at a temperature of 21°C and had *ad libitum* access to food and water. All the protocols of animal experiments were approved by the Animal Experimental Committee of Rosalind Franklin University of Medicine and Science (Protocol No. B21-07).

### Fecundity analysis

**Figure 1:**
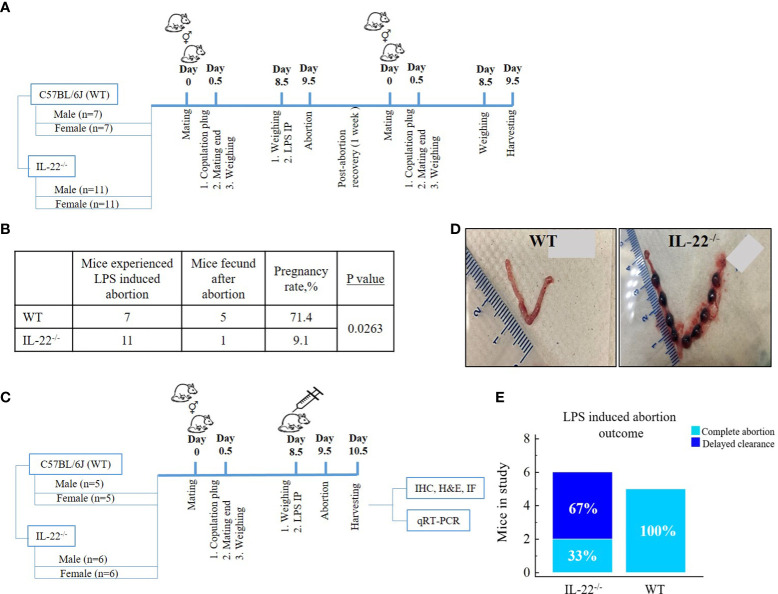
Mating schemes and outcomes. **(A)** Fecundity analysis: female mice were mated overnight with male mice. Females with copulation plug seen the next morning were designated as being on gestational day (gd) 0.5 of pregnancy, and further mating was over; 10µg LPS intraperitoneal injection was administered on gd 8.5. LPS-induced abortion was monitored and noted as the presence of blood in the cage on gd 9.5. After abortion, the same mice were mated again to check fecundity. **(B)** Fecundity table (outcome of chart A): WT mice have significantly higher fecundity rates in comparison to IL-22^−/−^ mice (t-test, p = 0.0263). **(C)** Mating to harvest experimental material: male and female mice were mated overnight and separated the next morning upon copulation plug observance. On gd 8.5, females were injected with intraperitoneal LPS endotoxin, monitored for abortion (gd 9.5), and harvested for future experiments (gd 10.5). **(D)** Delayed clearance after LPS-induced abortion in IL-22^−/−^ mice in comparison to WT mice that have complete abortion with clearance (WT, n = 5; IL-22^−/−^, n = 6). Shown are representative images of uteri harvested 48 h after LPS treatment on gd 8.5. **(E)** In the second mating scheme, IL-22^−/−^ mice demonstrated aborted fetuses that remained in the uterus, while WT mice had completed abortion with total clearance (chi-square test, p = 0.09). MedCalc software was used to create the table **(B)** and the bar chart **(E)**. LPS, lipopolysaccharide; WT, wild type; IL-22^−/−^, IL-22 knockout; IP, intraperitoneal; gd, gestational day.

Female mice were mated overnight with male mice of proven fertility (IL-22^−/−^, n = 11; WT, n = 7). Females with copulation plug observed the following morning were designated as gestational day (gd) 0.5 of pregnancy. Bacterial endotoxin LPS in the dose of 10 µg per mouse was administered as one intraperitoneal injection at gd 8.5. LPS-induced abortion was monitored and noted as the presence of blood in the cage or the presence of intact or partial fetal tissue (22) on gd 9.5. Following a recovery period of 1 week after the abortion, the mice were repeatedly mated. The mating scheme is presented in **Figure 1A**. Pregnancy success following repeated mating was recorded for fecundity analysis (**Figure 1B**).

### Material harvest

The second round of mating was performed with females that had not participated in previous mating and males of proven fertility (**Figure 1C**). One-time 10µg LPS intraperitoneal injection per mouse was administered at gd 8.5 to induce pregnancy loss. A total of n = 11 mice (WT, n = 5; IL-22^−/−^, n = 6) were harvested 48 h post LPS injection. Animals were euthanized with a CO_2_, and a gross examination of the uteri was performed. The presence of non-viable implantation sites was considered an incomplete abortion in contrast to a complete clearance, which was recorded as a complete abortion (**Figures 1D, E**). The uterine tissues were further processed for immunohistochemistry (IHC), hematoxylin and eosin staining, reverse transcription quantitative real-time PCR (RT-qPCR), and immunofluorescence (IF).

### Reagents used

The list of antibodies used in this study can be found in **Table 1**. Reagents used in the study are as follows: hematoxylin and eosin stain kit (Catalog No. H-3502, Vector Laboratories, CA, USA), Vectastain Elite ABC Universal PLUS kit (Catalog No. PK-8200, Vector Laboratories, CA, USA), Mouse on Mouse immunodetection kit (Catalog No. BMK-2202, Vector Laboratories, CA, USA), Opal™ 7-color manual IHC kit (Lot 210525137, Akoya Biosciences, MA, USA), biotinylated horse anti-rabbit IgG antibody (H+L) (Catalog No. BA-1100-1.5, Vector Laboratories, CA, USA), antigen unmasking solution, citrate-based (Catalog No. H-3300, Vector Laboratories, CA, USA), acetone (Lot No. 175113, Fisher Chemical, NH, USA), ethanol (CAS 64-17-5, Decon Laboratories, PA, USA), xylene (Catalog No. 534056-4L, Honeywell, NC, USA), 16% paraformaldehyde solution (w/v) (Lot No. WE328310, IL, USA), 10× phosphate-buffered saline (PBS) (Catalog No. AM9625, Thermo Fisher Scientific, MA, USA), hematoxylin (Catalog No. H-3401-500, Vector Laboratories, CA, USA), advanced pap pen (Daido Sangyo, Japan), Faramount aqueous mounting medium (Code S3025, Dako, CA, USA), ProLong™ diamond antifade mountant with 4′,6-diamidino-2-phenylindole (DAPI) (Lot 2080838, Invitrogen, MA, USA), TRIzol isolation reagent (Lot 350511, Ambion by Life Technologies, CA, USA), lipopolysaccharide from *Salmonella enterica* serotype Typhimurium (Lot 125M4123V, Sigma-Aldrich, MO, USA), and SuperScript IV reverse transcriptase (Lot 01099382, Invitrogen, CA, USA).

**Table 1:** Antibodies.

N	Antibody name	Manufacturer	Catalog number	Used concentration
				IHC/IF
1	MMP-2, rabbit polyclonal	Bioss, MA, USA	bs-4599R	1:1,000
2	MMP-3, rabbit polyclonal	Bioss, MA, USA	bs-0413R	1:1,000
3	MMP-9, rabbit polyclonal	Bioss, MA, USA	bs-4593R	1:1,000
4	Claudin-2, rabbit polyclonal	Novus Biologicals, CO, USA	NBP2-92140	1:2,000
5	Claudin-3, rabbit polyclonal	Invitrogen, MA, USA	34-1700	1:4,000
6	Claudin-10, rabbit polyclonal	Bioss, MA, USA	bs-13739R	1:4,000
7	Collagen-1, rabbit monoclonal	Abcam, Cambridge, UK	Ab270993	1:1,000
8	Collagen-3, rabbit polyclonal	Bioss, MA, USA	bs-0948R	1:1,000
9	Fibronectin, rabbit polyclonal	Bioss, MA, USA	bs-0666R	1:1,000
10	Mucin-1, rabbit monoclonal	Novus Biologicals, CO, USA	NBP2-62561	1:100
11	Cytokeratin-8, rabbit polyclonal	Bioss, MA, USA	bs-1106R	1:1,000
12	Pan-cytokeratin, FITC conjugated	Millipore Sigma, MA, USA	F0397	1:100
13	WNT-4, rabbit polyclonal	Bioss, MA, USA	bs-1106R	1:1,000
14	WNT-7A, rabbit polyclonal	Bioss, MA, USA	bs-6645R	1:1,000
15	E-cadherin, rabbit polyclonal	Bioss, MA, USA	bs-10009R	1:1,000
16	N-cadherin, rabbit polyclonal	Proteintech, IL, USA	22018-1-AP	1:4,000

MMP, matrix metalloproteinase; WNT, wingless-related integration site; IHC, immunohistochemistry; IF, immunofluorescence.

### Tissue preparation

Uterine tissue of IL-22^−/−^ (n = 6) or WT (n = 5) mouse was collected 48 h after the treatment with LPS. The tissue was fixed with 4% paraformaldehyde (24 h, room temperature), washed with PBS, dehydrated with 30% sucrose (24 h, room temperature), and frozen in Tissue-Tec O.C.T. (Sakura, CA, USA) above liquid nitrogen in cryo molds. It was kept at −80°C until further use.

### Immunohistochemical staining

The procedure was performed per the manufacturer’s recommendations using the Vectastain Elite ABC Universal PLUS kit. Briefly, the frozen tissue blocks were brought to −20°C, and 5µm sections were cut with a cryostat (Leica, Mannheim, Germany) and placed on slides (Fisherbrand, Tissue path Superfrost Plus Gold, PA, USA). After fixing in acetone, unmasking the antigens, quenching the endogenous peroxidase activity in BLOXALL blocking solution for 10 min, and incubating in normal horse serum (Vectastain Elite ABC Universal PLUS kit component), the samples were probed overnight at −4°C with primary antibodies or horse anti-rabbit IgG diluted in PBS. The following day, the slides were washed three times in PBS and incubated for 1 h at room temperature with goat anti-rabbit IgG antibodies and Vectastain Elite ABC reagent. Bound antibodies were then visualized using 3,3′-diaminobenzidine (DAB), the slides were counterstained with hematoxylin, and staining was assessed under a Leica ICC50W microscope. The histological score (H-score) was calculated by assigning a 4-point scale to the intensity of the staining in 10 random fields (0 indicates negative; 1+, weak; 2+, moderate; and 3+, strong). The percentage of cells at each intensity level was calculated, and H-score was assigned using the following formula: [1 × (% cells, 1+) + 2 × (% cells, 2+) + 3 × (% cells, 3+)]. The final score, ranging from 0 to 300, is considered to be strongly positive (201–300), moderately positive (101–200), weakly positive (1–100), or negative (0) (23).

### Hematoxylin and eosin staining and cell density evaluation

Frozen sections (5 µm) were cut with a cryostat and placed on slides. After being fixed with 100% acetone for 5 min and dried at room temperature, the slides were exposed to deionized water, hematoxylin (1:4 dilution), bluing reagent, and eosin consecutively, followed by stepwise dehydration, cleaning, and mounting procedures. The slides were visualized under a Leica ICC50W microscope. Cell density was determined using the ImageJ software application with the CellProfiler option for Windows (NIH, 1.53n/2021; github.com/imagej/imagej1).

### Immunofluorescence

Multiplexed IF staining was performed using Opal kit as per the manufacturer’s protocol and as described previously (23). Briefly, 5µm frozen sections were exposed to epitope retrieval, blocked, and incubated with primary antibody (1 h at room temperature), and then horseradish peroxidase and the secondary antibody were introduced. After the signal amplification with fluorophores, the slides were counterstained with DAPI, and mounted using ProLong™ diamond antifade mountant. Then the coverslipped sections were examined by confocal microscopy and imaged on Olympus Fluoview Fv10i confocal microscope. Analysis was performed using fv10i flouview Ver.3.0 software (Olympus Europa Holding GmbH, Hamburg, Germany). For IF microscopy, stained tissue was imaged by Olympus microscope and analyzed using NIS-Elements Ar software (Nikon Inc., NY, USA).

### RNA extraction and RT-qPCR

Total RNA was extracted from uterine tissue using a TRIzol isolation reagent. Non-cleared fetal tissue was removed during tissue harvest to ensure that only maternal uterine tissue was used for RNA extraction. Sample quality check was performed using NanoDrop spectrophotometer as per manufacturer’s instructions. Oligo-primed cDNA was synthesized using SuperScript IV reverse transcriptase. Primer-BLAST, NCBI’s free online primer design platform, was used to design PCR primers by the Primer3 (https://www.ncbi.nlm.nih.gov/tools/primer-blast/) (24). The primers were designed for MMP-2, MMP-3, MMP-9, claudin-2, claudin-3, claudin-10, Collagen-1a1, Collagen-3a1, Fibronectin-1, Mucin-1, Cytokeratin-8, WNT-4, WNT-7, E-cadherin, N-cadherin, and GADPH (housekeeping gene). All forward and reverse primers used in the study are listed in **Table 2**. PCR was carried out on a Step One Plus™ Real-Time PCR system Thermal Cycling Block (Applied Biosystems by Thermo Fisher Scientific, Waltham, MA, USA) using SYBR Green Master Mix (Applied Biosystems by Thermo Fisher Scientific, Waltham, MA, USA) following the manufacturer’s instructions. The expression levels of all genes of interest are taken relative to GAPDH.

**Table 2:** Primers used in the study.

N	Name	Sequences: forward and reverse	N	Name	Sequence
1	GAPDH	5′-TGTGAACGGATTTGGCCGTA-3′, 5′-ACTGTGCCGTTGAATTTGCC-3′	9	Collagen-3	5′-GAGGAATGGGTGGCTATCCG-3′ 5′-TCGTCCAGGTCTTCCTGAC-3′
2	MMP-2	5′-AAACAAGGCTTCATGGGGGC-3′, 5′-AACGGTCGGGAATACAGCAG-3′	10	Fibronectin-1	5′-GACACGTGGAGCAAGAAGGA-3′ 5′-TGTCGCTCACACTTCCACTC-3′
3	MMP-3	5′-TGCATGACAGTGCAAGGGAT-3′ 5′-ACACCACACCTGGGCTTATG-3′	11	Mucin-1	5′-CCAAGCGTAGCCCCTATGAG-3′ 5′-GTGGGGTGACTTGCTCCTAC-3′
4	MMP-9	5′-CGACTTTTGTGGTCTTCCCC-3′ 5′-CTTCTCTCCCATCATCTGGGC-3′	12	Cytokeratin-8	5′-TATGGGGGACTCACTAGCCC-3′ 5′-CAGCTTCCCATCTCGGGTTT-3′
5	Claudin-2	5′-AAGGACGGCTCCGTTTTCT-3′ 5′-ATCTTCGGAGCCTGTTTGCT-3′	13	WNT-4	5′-CAGAACACCAGCCAGACTGT-3′ 5′-GTGGAAGCATCGAACCTGGA-3′
6	Claudin-3	5′-GACCGTACCGTCACCACTAC-3′ 5′-CAGCCTAGCAAGCAGACTGT-3′	14	WNT-7	5′-CCCTTGTTGCGCTTGTTCTC-3′ 5′-GGGGCAATCCACATAGCCTG-3′
7	Claudin-10	5′-CTGGAAGGTCTCCACCATCG-3′ 5′-TAACCATCCAACGCCAGCAT-3′	15	E-cadherin	5′-CTCGCCCTGCTGATTCTGAT-3′ 5′-TTTCGAGTCACTTCCGGTCG-3′
8	Collagen-1	5′-CGACCTCAAGATGTGCCACT-3′ 5′-CCATCGGTCATGCTCTCTCC-3′	16	N-cadherin	5′-TCCAAGTGGCCAGGAAACGG-3′ 5′-GTGCGACAAAGCTTCCGGG-3′

GAPDH, glyceraldehyde-3-phosphate dehydrogenase; MMP, matrix metalloproteinase; WNT, wingless-related integration site.

### Statistical analysis

All statistical analyses were performed by using the MedCalc (MedCalc software, version 13, Belgium) statistical software. To compare the difference in the means between groups, two-tailed Student’s t-test and the Mann–Whitney U-test were used. Categorical data were analyzed by a chi-square test for trends. The differences were considered to be statistically significant when p < 0.05. Data are graphically represented as mean ± standard error of the mean (SEM). All experiments were performed in duplicate at least three times.

## Results

### Fecundity in IL-22^−/−^ mice and the importance of IL-22 in post-abortion clearance of fetuses

Since IL-22 is known to play an important role in tissue regeneration after an injury (16), we hypothesized that the absence of IL-22 might have an effect on the proper recovery of the endometrium following an LPS-triggered pregnancy loss in IL-22^−/−^ mice. In our fecundity analysis, female mice were mated twice (**Figure 1A**). A pregnancy that was achieved with the first mating was terminated by LPS treatment on gd 8.5, and the animals were allowed to recover before the second set of matings. We found that the rate of pregnancy success after the second mating was substantially different between IL-22^−/−^ and WT mice (**Figure 1B**), while there was no difference between the groups on initial mating (data not shown). IL-22^−/−^ mice revealed a significantly reduced ability to become pregnant (9.1% of pregnancy success) in comparison to WT mice (71.4% of pregnancy success) (**Figure 1B**), suggesting that IL-22 deficiency might be associated with secondary infertility. Among eleven IL-22^−/−^ mice, there was one mouse that managed to become pregnant following recovery after LPS-induced abortion. It is noteworthy that this mouse had implantation sites that were small for gestational age (**Supplementary Figure 1**). Moreover, a considerable difference was observed between IL-22^−/−^ and WT mice in the uterine clearance following LPS treatment and pregnancy loss (**Figure 1C**). Gross examination of the uteri of IL-22^−/−^ mice revealed retained non-viable fetuses inside the horns (delayed clearance) (**Figure 1D**). Among six IL-22^−/−^ mice, an incomplete abortion was recorded in four animals (**Figure 1E**). In contrast, all WT mice had completed abortion with total clearance after LPS exposure (**Figure 1D**), leading us to further analyze the role of IL-22 in endometrial homeostasis and regeneration.

### The role of IL-22 in extracellular matrix organization

**Figure 2:**
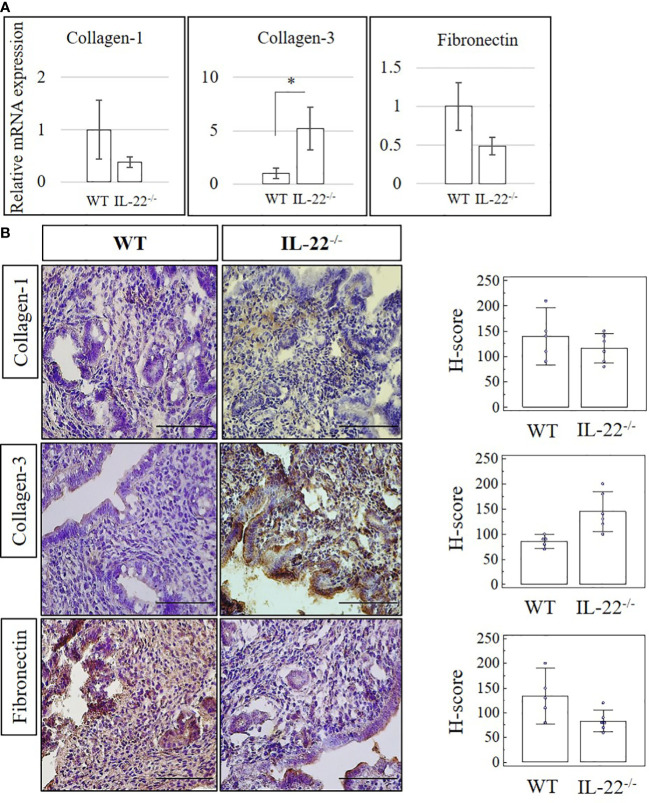
The expression of extracellular matrix proteins in IL-22^−/−^ and WT mice. **(A)** IL-22^−/−^, n = 6, and WT, n = 5, mouse uterine samples were harvested 48 h post intraperitoneal LPS injection. Total mRNA samples were extracted and processed by qRT-PCR to evaluate the expression of Collagen-1, Collagen-3, and fibronectin. The data are provided as mean ± SEM from three independent experiments. **(B)** IL-22^−/−^, n = 6, and WT, n = 5, mouse uterine samples were harvested 48 h post intraperitoneal LPS injection, frozen fixed, and processed for IHC analysis. Original magnification, ×40; scale bar, 100 µm. H-score of ECM proteins was calculated to measure whether the difference in the staining between the groups is statistically significant. The data from the t-test are provided as mean ± SEM. WT, wild type; IL-22^−/−^, IL-22 knockout; H-score, histological score; LPS, lipopolysaccharide; IHC, immunohistochemistry; ECM, extracellular matrix. * p < 0.05.

Proteolytic enzymes contribute to epithelial regeneration *via* extracellular matrix (ECM) reconstruction after menstruation, postpartum, or abortion (1). We compared the expression of matrix metalloproteinases (MMP-2, MMP-3, and MMP-9) in uterine tissues between WT and IL-22^−/−^ mice after LPS-induced abortion. While IL-22^−/−^ mice, overall, were found to have a higher mRNA expression of MMPs in comparison to WT mice, the mRNA expression did not reach a statistical significance (**Supplementary Figure 2A**). IHC staining confirmed RT-qPCR findings with no significance between WT and IL-22^−/−^ in histological scores (H-score) (**Supplementary Figure 2B**). Therefore, we can conclude that IL-22 is not essential in the recruitment of matrix metalloproteinase to repair the endometrium after LPS-induced abortion. Collagen-3 was significantly more abundant in IL-22^−/−^ mice on both mRNA and protein levels (**Figures 2A, B**). Other fibrous components of ECM, like Collagen-1, Fibronectin (**Figures 2A, B**), and Cytokeratin-8 (**Supplementary Figure 3**), were not significantly different between WT and IL-22^−/−^ mice. H-score was applied to quantify the difference in staining between the groups and showed a significant upregulation of Collagen-3 protein in IL-22^−/−^ mice (**Figure 2B**), suggesting that IL-22 might be important to limit the excess amount of ECM components in the regenerative process.

### IL-22 affects epithelial permeability *via* modulation of tight junctions

**Figure 3:**
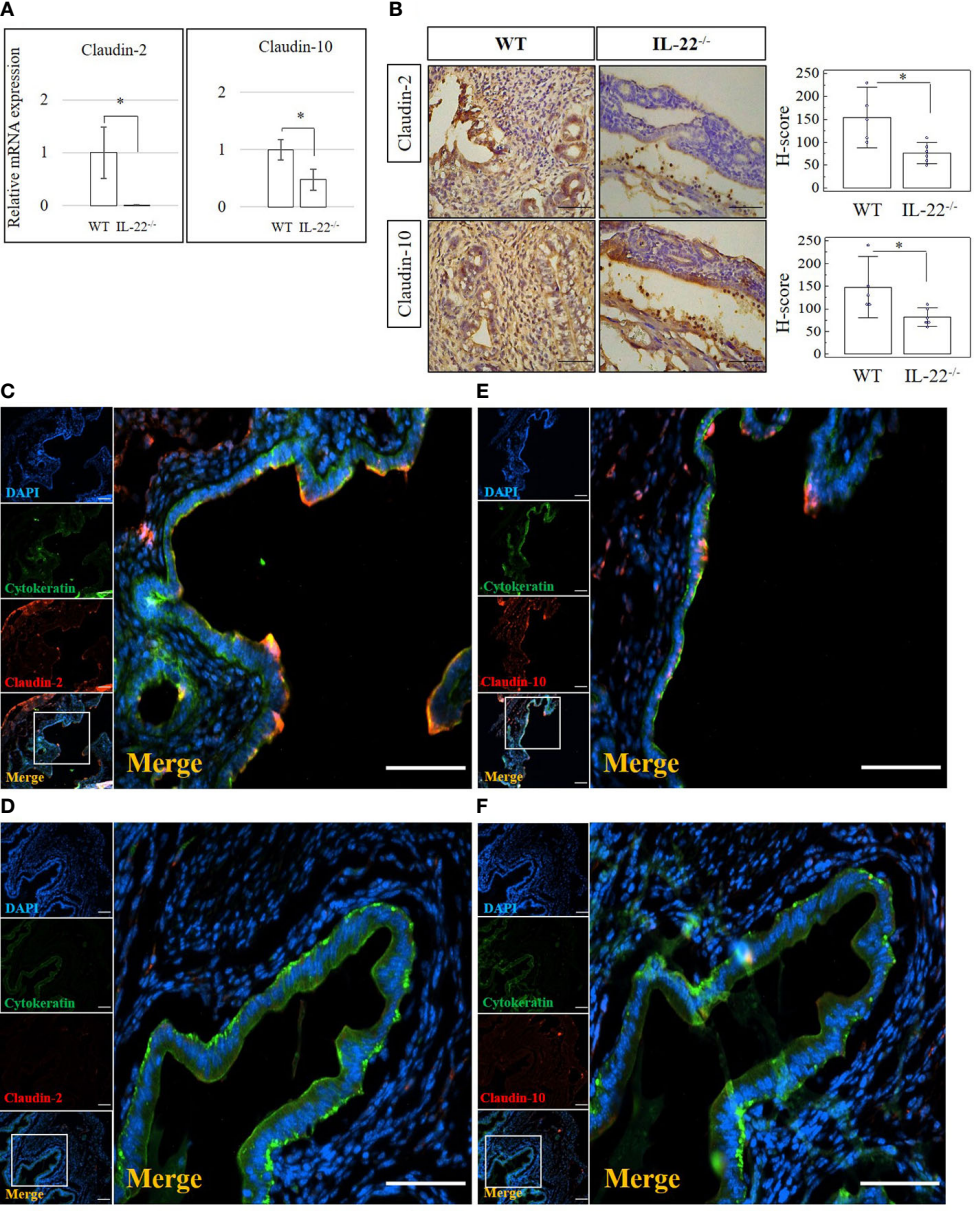
The expression of tight junction molecules in IL-22^−/−^ and WT mice. **(A)** IL-22^−/−^, n = 6, and WT, n = 5, mouse uterine samples were harvested 48 h post intraperitoneal LPS injection. Total mRNA samples were extracted and processed by qRT-PCR to quantify the expression of tight junction molecules. The data are provided as mean ± SEM from three independent experiments. **(B)** IL-22^−/−^, n = 6, and WT, n = 5, mouse uterine samples were harvested 48 h post intraperitoneal LPS injection, frozen fixed, and processed for IHC analysis. Original magnification, ×40; scale bar, 100 µm. H-score of tight junctions was calculated to measure whether the difference in the staining between the groups is statistically significant. The data from t-test are provided as mean ± SEM. **(C–F)** IF analysis of claudins’ expression in WT **(C, E)** and IL-22^−/−^ mice **(D, F)**. Claudin-2 staining is shown in panels **(C**, **D)** and claudin-10 staining in panels **E, F** Small panels on the left demonstrate (from top to bottom) the following: nuclei staining in DAPI, blue; pan-cytokeratin staining in FITC, green; claudin-2 or claudin-10 in TRITC, red; merged image. Original magnification, ×10. Large panels demonstrate insets shown on the merged images; original magnification, ×20. Scale bar is 100 µm. WT, wild type; IL-22^−/−^, IL-22 knockout; H-score, histological score; LPS, lipopolysaccharide; IHC, immunohistochemistry; IF, immunofluorescence; FITC, fluorescein isothiocyanate. * p < 0.05.

Tight junctions, including claudins, are essential for the continuous intercellular barrier between epithelial cells, which is required to separate tissue spaces and regulate the selective movement of solutes across the epithelium (4). We evaluated the expression of claudin-2, claudin-3, and claudin-10 in the uterine samples from IL-22^−/−^ and WT mice after LPS-triggered abortion. From our data, IL-22^−/−^ mice have a significantly decreased mRNA expression of both claudin-2 and claudin-10 (**Figure 3A**). Claudin-3 was less expressed in IL-22^−/−^ in comparison to WT mice, but the difference did not reach a statistical significance between the groups (**Supplementary Figure 4A**). Protein levels of claudins were evaluated by immunohistochemical analysis (**Figure 3B**). A significantly lower expression of claudin-2 and claudin-10 was found in IL-22^−/−^ mice in comparison to WT controls (**Figure 3B**). However, there was no significant difference for claudin-3 staining between the groups that were evaluated by applying H-score (**Supplementary Figures 4B, C**). IF analysis revealed that WT uterine luminal epithelium expresses both claudin-2 (**Figure 3C**) and claudin-10 (**Figure 3E**), while only claudin-10 was detected on the glandular epithelium (**Figure 3E**). In contrast, very faint staining if any was observed for claudin-2 (**Figure 3D**) and claudin-10 (**Figure 3F**) in uterine tissues from IL-22^−/−^ animals. Quantification of immuno-fluorescent intensities of claudins’ staining was performed on ImageJ software (**Supplementary Figure 5A**).

**Figure 4:**
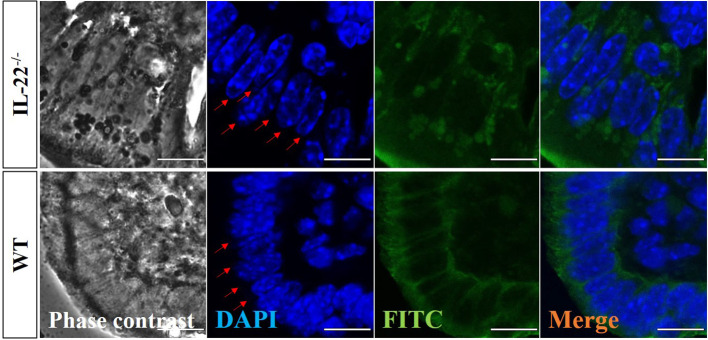
Confocal microscopy evaluation of uterine cross-sections. IL-22^−/−^, n = 6, and WT, n = 5, mouse uterine samples were harvested 48 h post intraperitoneal LPS injection, frozen fixed, and processed for IF analysis. Representative images demonstrate phase-contrast (left column), DAPI (middle left column), pan-cytokeratin (middle right column), and merged (right column) images. Red arrows point to the nuclei of luminal epithelium (from the lumen side) to demonstrate the distinct structural organization with epithelial cells forming a uniform lining in WT and the pseudo-stratified appearance with epithelial cell nuclei located at different heights in IL-22^−/−^ samples. Original magnification 60 × 5; scale bar, 10 µm. WT, wild type; IL-22^−/−^, IL-22 knockout; LPS, lipopolysaccharide; IF, immunofluorescence.

A z-stack analysis using confocal microscopy on uterine cross-sections from animals that experienced the LPS-induced abortion also showed lower expression of both claudin-2 and claudin-10 in IL-22^−/−^ mice and provided a precise understanding of the localization of tight junctions (**z-stack of claudin-2, z-stack of claudin-10, mp4**). Both claudin-2 and claudin-10 were localized on the apical and uppermost parts of lateral membranes of the luminal epithelial cells in WT and IL-22^−/−^ mice. Following abortion, the restored luminal epithelial layer in samples from IL-22^−/−^ mice revealed a pseudo-stratified appearance when epithelial cell nuclei are located at different heights (**Figure 4**). In contrast, in WT samples, the luminal epithelium had a distinct structural organization with epithelial cells forming a uniform lining (**Figure 4**). The difference in epithelial layer morphology was assessed using the region of interest function of fv10i flouview Ver.3.0 software (**Supplementary Figures 5B–E**). Hence, IL-22^−/−^ mice have impaired epithelial permeability due to the insufficient amount of tight junction molecules that were supposed to regulate the flux of sodium, water, and cellular volume. In the endometrium, “endometrial leakage” is probably a period of increased secretion, a potentially important mechanism in LPS-induced pregnancy loss, as it prepares the endometrium for the following pregnancy or normal function by tissue remodeling. Thus, the absence of IL-22 could lead to abnormal re-epithelization of the endometrium after the LPS challenge.

### IL-22 is a critical regulator of epithelial homeostasis

**Figure 5:**
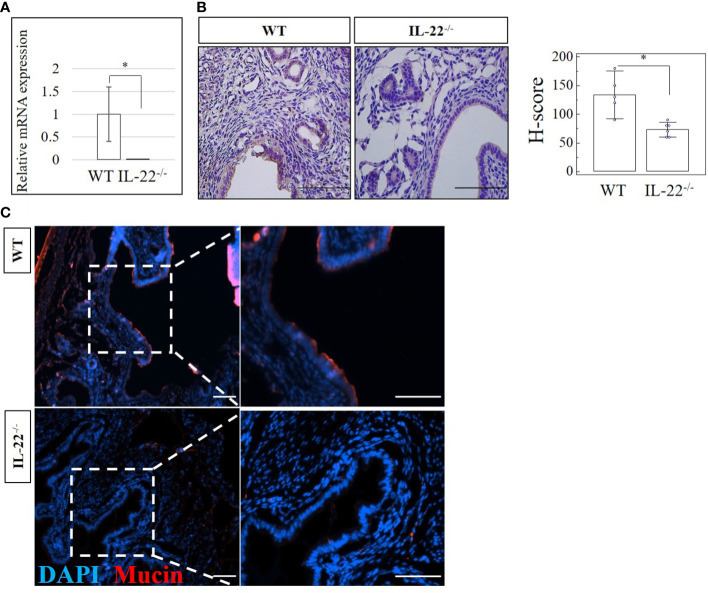
The expression of a cell surface pathogen protector mucin-1 in IL-22^−/−^ and WT mice. **(A)** IL-22^−/−^, n = 6, and WT, n = 5, mouse uterine samples were harvested 48 h post intraperitoneal LPS injection. Total mRNA samples were extracted and processed by qRT-PCR to evaluate the expression of mucin-1. The data are provided as mean ± SEM from three independent experiments. **(B)** IL-22^−/−^, n = 6, and WT, n = 5, mouse uterine samples were harvested 48 h post intraperitoneal LPS injection, frozen fixed, and processed for IHC analysis. Original magnification, ×40; scale bar, 100 µm. H-score of mucin-1 was calculated to measure whether the difference in the staining between the groups is statistically significant. The data from t-test are provided as mean ± SEM. **(C)** Representative IF images of mucin-1 in IL-22^−/−^ and WT mice. Nuclei staining in DAPI, blue; mucin-1 in TRITC, red. Original magnification, ×10 and ×20; scale bar, 100 µm. WT, wild type; IL-22^−/−^, IL-22 knockout; H-score, histological score; LPS, lipopolysaccharide; IHC, immunohistochemistry; IF, immunofluorescence. * p < 0.05.

IL-22 is involved in many aspects of epithelial barrier function like regulation of epithelial permeability and production of antimicrobial proteins in different areas of the body. Mucins produced by mucosal tissue play an important role in the defense against pathogens due to their ability to form a molecular barrier (25). In our study, IL-22^−/−^ mice have significantly reduced amounts of mucin-1 mRNA and protein when compared to WT mice (**Figures 5A, B**). H-score proved the significance of the immunohistochemical findings (**Figure 5B**), suggesting that IL-22 is important in cell surface pathogen protectors’ upregulation. To further validate this finding, the expression of mucin-1 was assessed by IF and confocal microscopy analysis. IF revealed significantly higher expression of mucin-1 in WT mice than in IL-22^−/−^ mice (**Figure 5C**), while confocal analysis provided a detailed localization of mucin-1 (it is expressed on the apical part of luminal epithelial cells) and confirmed IF findings (**z-stack of mucin-1, mp4**). Quantification of immuno-fluorescent intensities of mucin-1 staining was performed on ImageJ software (**Supplementary Figure 5A**).

### IL-22 is necessary for adherens junctions’ regulation

**Figure 6:**
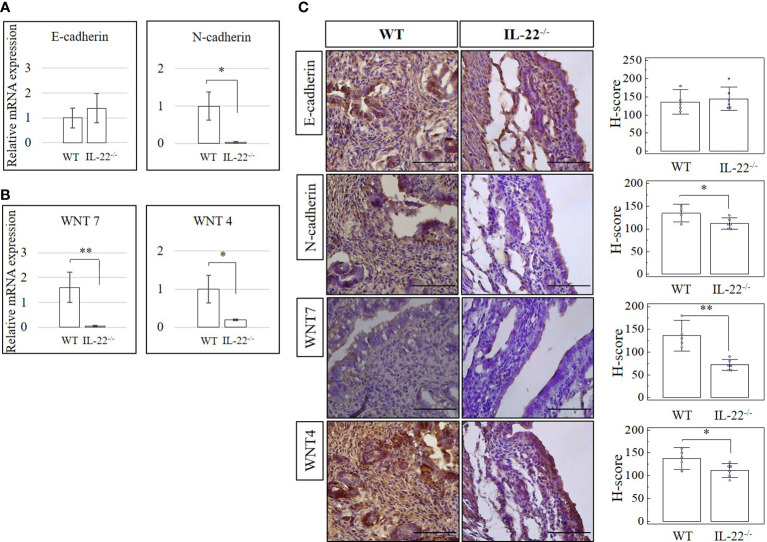
The expression of cadherin and WNT molecules in IL-22^−/−^ and WT mice. **(A, B)** IL-22^−/−^, n = 6, and WT, n = 5, mouse uterine samples were harvested 48 h post intraperitoneal LPS injection. Total mRNA samples were extracted and processed by qRT-PCR to evaluate the expression of cadherin and WNT molecules, respectively. The Ct values were normalized against the Ct values for GAPDH from the same preparation. The data are provided as mean ± SEM from three independent experiments. **(C)** IL-22^−/−^, n = 6, and WT, n = 5, mouse uterine samples were harvested 48 h post intraperitoneal LPS injection, frozen fixed, and processed for IHC analysis. H-score of cadherin and WNT molecules was calculated to measure whether the difference in the staining between the groups is statistically significant. The data from t-test are provided as mean ± SEM. WT, wild type; IL-22^−/−^, IL-22 knockout; H-score, histological score; WNT, wingless-related integration site; LPS, lipopolysaccharide; IHC, immunohistochemistry. Original magnification, ×40; scale bar, 100 µm. * p < 0.05.

Adherens junctions composed of complexes of E-cadherin and N-cadherin and WNT signaling pathway molecules are commonly used to recognize the transitioning between epithelial and mesenchymal cells (26). Epithelial–mesenchymal transition (EMT) can be defined by the activation of the mesenchymal and the decrease of the epithelial cell characteristics (27). Alternatively, upregulation of E-cadherin and downregulation of N-cadherin could be indicative of a mesenchymal–epithelial transition (MET) (26, 27). We checked uterine tissues from IL-22^−/−^ and WT mice after the LPS-triggered abortion for N-cadherin, E-cadherin, WNT-4, and WNT-7 expression on mRNA (RT-qPCR) and protein (IHC and H-score) levels. A significantly lower expression of N-cadherin on both mRNA and protein levels was observed in IL-22^−/−^ mice than in WT mice (**Figures 6A–C**). There was no significant difference between groups of E-cadherin mRNA or protein levels when H-scores were applied (**Figures 6A–C**). However, it was obvious that both WNT-4 and WNT-7 were significantly less expressed in IL-22^−/−^ mice in comparison to WT mice.

### IL-22 can account for cell density and stromal homeostasis

**Figure 7:**
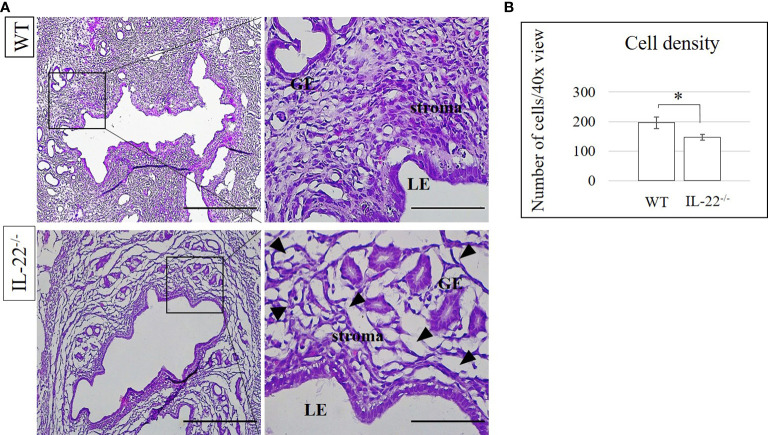
The density of cells in IL-22^−/−^ and WT mice. **(A)** IL-22^−/−^, n = 6, and WT, n = 5, mouse uterine samples were harvested 48 h post intraperitoneal LPS injection, frozen fixed, and processed for H&E analysis. Black arrowheads are pointing at the spaces in uterine stroma (“stromal edema”). **(B)** Cell density was calculated by Cell Counter Plugin in ImageJ software to measure whether the difference between the groups is statistically significant. The data from t-test are provided as mean ± SEM. WT, wild type; IL-22^−/−^, IL-22 knockout; LPS, lipopolysaccharide. Original magnification, ×10 and ×40. Scale bar, 100 µm. * p < 0.05.

After performing hematoxylin and eosin staining for tracing any histo-morphological changes in WT and IL-22^−/−^ uterine tissues, we measured cell densities to find out whether there were any differences between the groups. The stroma of IL-22^−/−^ mice has a lower number of cells in the areas of the luminal and glandular epithelium in comparison to that of WT mice (**Figure 7A**). The difference achieved was statistically significant, confirming the difference in the density of the stromal compartment in IL-22^−/−^ and WT mice (**Figure 7B**). The lower number of stromal cells can be due to stromal edema that occurred in IL-22-deficient mice post endotoxin-induced abortion.

## Discussion

IL-22 is detected in various tissues, including the liver, skin, thymus, lung, pancreas, gastrointestinal tract, synovium, heart, and adipose tissue, and plays an important immunoregulatory role (14). Its release accomplishes immediate regeneration with inflammation mediation, mucous production, and pathogen protection (16). However, an excessive increase in these activities leads to carcinogenesis (28, 29) as well as psoriasis and other immune diseases (14). Mediating pro- and anti-inflammatory responses, IL-22 can exert both protective and pathological functions. The reports published about IL-22 in uterine pathobiology indicate that IL-22 is necessary for trophoblast survival in early gestation (30), LPS-induced preterm labor prevention in late gestation (21), and endometrial homeostasis and inflammation prevention throughout pregnancy (31). It was reported that in contrast to normal pregnancies, spontaneous miscarriages have reduced levels of IL-22/IL-22R1 in placental villi (30). Another study involving patients with unexplained RPL and unexplained infertility concludes that RPL patients have significantly higher endometrial IL-22 levels than healthy controls (31). A recent study shows that direct intra-amniotic IL-22 injection can cause fetal injury and neonatal death, but an elevated concentration of IL-22 in amniotic fluid is necessary for host defense in microbial invasion often found in women with preterm labor (32).

There has been limited information published about the role of IL-22 in uterine regeneration. This work demonstrates that IL-22 is key for the proper regeneration of endometrial layers after inflammation-triggered abortion. The data show that IL-22 enhances tight junctions (claudin-2 and claudin-10) and upregulates cell surface pathogen protectors (mucin-1). We also discovered that IL-22 contributes to the remodeling of the uterine tissue in the inflammatory environment by regulating epithelial–mesenchymal and mesenchymal–epithelial transition markers (N-cadherin, E-cadherin, WNT-7, and WNT-4).

Knowing that IL-22 participates in tissue repair after an injury (16), we hypothesized that the absence of IL-22 might hinder endometrial regeneration after LPS-triggered abortion in IL-22^−/−^ mice and affect the mice’s ability to become pregnant in subsequent matings. Indeed, our IL-22^−/−^ mice have significantly lower fecundity rates in consecutive matings after LPS-induced abortion on gd 8.5, although they became successfully pregnant for the first time. Even if IL-22^−/−^ mice manage to become pregnant, their implantation sites are smaller than in WT mice. This can be explained by the absence of IL-22 and its necessity to prevent secondary infertility in IL-22-deficient mice as shown above. Stroma-derived epithelium transiently fills the gaps in resident epithelial cells in regeneration after birth (33). In our fecundity analysis, this might delineate why IL-22-deficient mice can handle the first pregnancy and fail to maintain consecutive pregnancies, as the absence of IL-22 might lead to the inability of stromal cells to transition into epithelial tissue in a second pregnancy. These findings can be attributed to the role of IL-22 in cell proliferation and proper re-epithelization (34).

Re-epithelization heavily depends on ECM (35). Collagen, fibronectin, and cytokeratin are the most abundant fibrous proteins in ECM that provide tensile strength, regulate cell adhesion, support chemotaxis and migration, and direct tissue development (36). Depending on the amount, ECM proteins exert pleiotropic effects including the promotion of wound healing, tumor growth, and metastasis (37). IL-22 is known to induce and regulate ECM production (37). Thus, IL-22 deficiency was associated with excessive ECM production during wound repair in the skin (38). This could contribute to abnormal healing that destroys tissue architecture and interferes with organ function. Proteolytic enzymes, like MMP-2, MMP-3, and MMP-9, limit the excessive production of ECM genes (39). Some studies infer that LPS and certain cytokines like IL-10 and IFN-γ might induce the production of MMPs (40–42). Our data suggest that IL-22 plays an important role in controlling the excessive production of ECM in uterine regenerative process after abortion. IL-22^−/−^ mice produced an increased amount of Collagen-3 compared to our controls. The regulation of proteolytic enzymes in endometrial repair after LPS-induced abortion is IL-22 independent, as the difference between the groups was not significant.

Cell–cell contact molecules have a wide variety of functions like influencing tissue physiology, homeostasis, and remodeling by controlling para- and intercellular transport, affecting signaling pathways, and maintaining cell plasticity *via* controlling its polarity (4). These direct cell–cell interactions are important because tight and adherens junctions, desmosomes, and gap junctions are localized on the lateral membranes of polarized epithelial cells (4). Claudin proteins constitute the largest family among tight junctions and are involved in proliferation and cell differentiation (43). In the endometrium, claudins show various expression patterns in physiology and pathology. Claudin-6 and claudin-4, which are absent in normal adult samples, are aberrantly expressed in endometrial cancer (44, 45). Downregulation of claudin-3, claudin-4, and claudin-7 may contribute to the establishment of endometriosis (46). Claudin-1, claudin-3, and claudin-5 restrict the passive movement of fluid to maintain the uterine luminal content surrounding developing embryos (47). In inflammatory intestinal diseases, claudin-2 is necessary to resolve epithelial barrier dysfunction by increasing its permeability for ions. IL-22 upregulates claudin-2 (48). In our study, we found that the absence of IL-22 resulted in abnormal re-epithelization of the endometrium after the LPS challenge. Therefore, in the endometrium, “endometrial leakage” might be a period of increased secretion with high permeability—a potentially important mechanism in LPS-induced pregnancy loss—as it prepares the endometrium for the following pregnancy or normal tissue remodeling.

Activation of endometrial epithelial progenitor cells and perivascular mesenchymal stem cells, involving the activation of WNT molecules and complex forming between WNT and adherens junctions (cadherins), mediates regeneration of the endometrium (49). An increase of E-cadherin and a decrease of N-cadherin, as well as an increase in WNT-4 and a decrease in WNT-7, are two main shifts that define the MET process (49). The IL-22^−/−^ mice in our study have increased E-cadherin and significantly less N-cadherin in comparison to WT mice but may lack proper complex forming between cadherins and WNT molecules, as IL-22^−/−^ mice have significantly decreased amounts of both WNT-4 and WNT-7. Our data demonstrate that the endometrium in IL-22^−/−^ mice can be undergoing an intermediate process between the mesenchymal–epithelial and epithelial–mesenchymal transition, while in WT mice, the uterus is already in an epithelial–mesenchymal state and ready for future pregnancies.

It has been reported that IL-22 induces the production of innate antimicrobial molecules, like defensins and mucins, to protect the epithelial barrier against invading pathogens (18). A reduction of IL-22 in the intestine increases the number of pathogenic bacteria in the gut. IL-22 can also drive polycystic ovary syndrome (PCOS) associated with ovarian and metabolic dysfunctions (50). With the use of a PCOS-like murine model, it was shown that treatment with more IL-22 could lead to a full recovery of ovarian morphology, reproductive cycles, and insulin resistance (50). According to our results, uterine tissues from IL-22-deficient mice that experienced LPS-triggered abortion have significantly lower levels of an antimicrobial protein called mucin-1 than tissues from WT mice. In line with previous findings, in our study, IL-22 is pivotal in upregulating cell surface pathogen protectors. While IL-22^−/−^ mice that lack mucin-1 have a hard time clearing the aborted fetuses after LPS-induced pregnancy loss, the WT counterparts have successfully undergone total clearance after LPS treatment.

**Figure 8:**
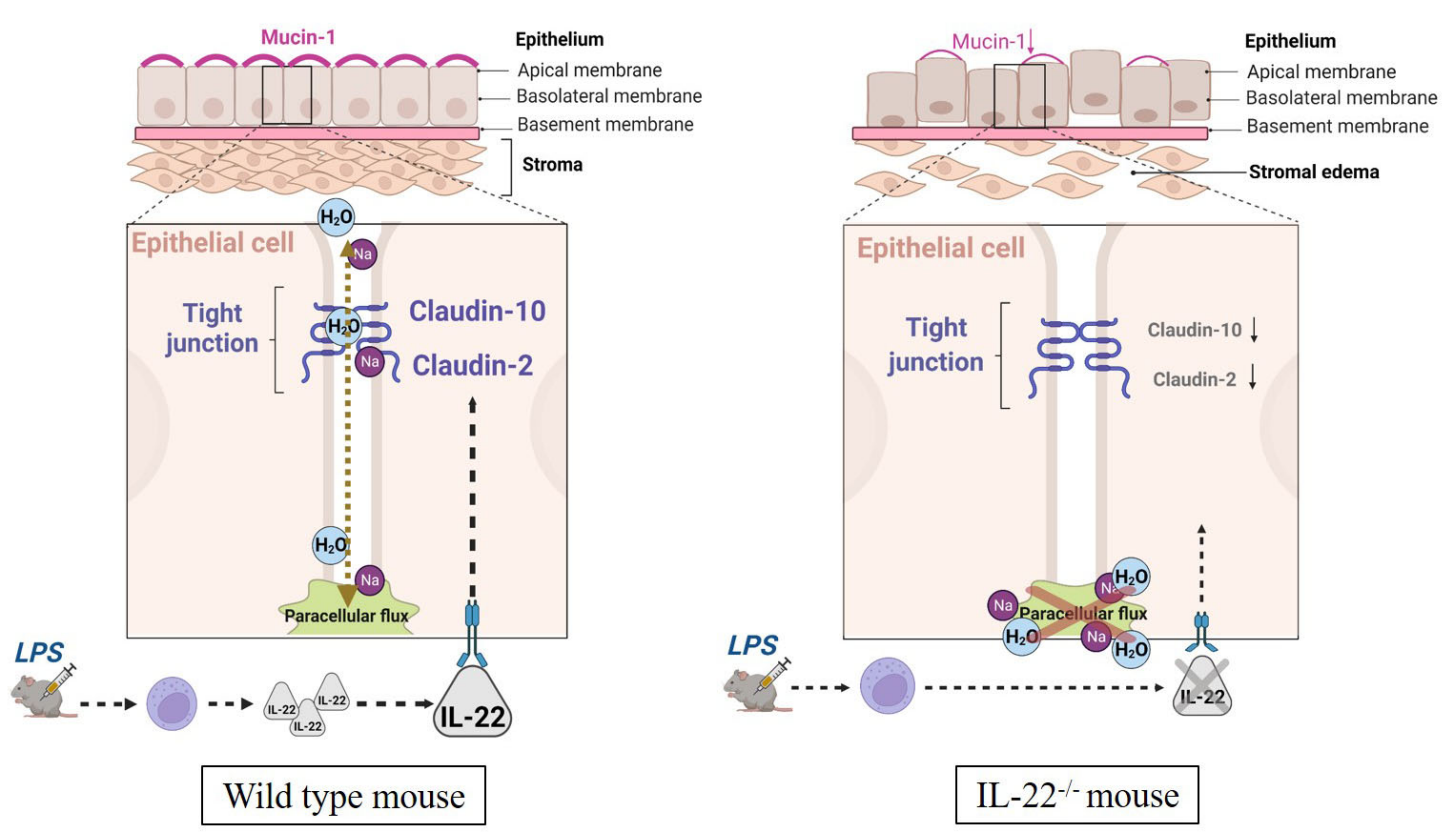
The schematic presentation of the impact of IL-22 on endometrial regeneration. In comparison to WT animals, IL-22^−/−^ mice have lower expression of tight junctions and mucin-1. Decreased amount of tight junctions leads to permeability reduction. Cellular density of stromal compartment in IL-22^−/−^ mice is lower than in WT mice. This might be explained by disrupted permeability of tight junctions, which fail to adequately facilitate paracellular flux (shown in green dotted double-headed arrow in WT mice). This might lead to stromal edema that occurred in IL-22^−/−^ mice post endotoxin-induced abortion. Hence, IL-22 enhances paracellular flux performed by tight junctions (claudin-2 and claudin-10) and upregulates cell surface pathogen protectors (mucin-1) contributing to proper regeneration of endometrial layers after inflammation-triggered abortion. IL-22 also participates in the remodeling of the uterine tissue in inflammatory environment by regulating other cell–cell contacts called adherens junctions (E- and N-cadherin; not shown on the scheme). Therefore, IL-22 can be considered to perform a dual role: enhance uterine regeneration postpartum and barrier/protect against secondary infertility caused by inflammation. LPS, lipopolysaccharide; WT, wild type; IL-22^−/−^, IL-22 knockout; Na, sodium; H_2_O, water.

Tight junctions, including claudins, create controlled paracellular movement of micro- and macromolecules between epithelial cells, thus maintaining epithelial barrier integrity and homeostasis. Barrier dysfunction with inadequate permeability of solutes and water across the membranes may result in capillary leak, pulmonary edema, and multiple organ failure (10). In the uterus, intrauterine fluid resorption is necessary for establishing uterine receptivity and preparing it for future pregnancies, whereas failure to resorb the fluids results in reduced embryo implantation and pregnancy rate (51). The IL-22^−/−^ mice in our study have less dense stroma in comparison to WT mice and have increased fluid retention that can occur as a result of the deficiency of claudins in knockout mice (51). In the absence of IL-22, claudins fail to control the appropriate transport of water, which results in stromal edema and leads to inappropriate remodeling of the uterine tissue after bacterial endotoxin-induced abortion in IL-22^−/−^ mice (**Figure 8**).

Among the limitations of this study is a lack of mechanistic data to confirm that the decrease in permeability is caused by the disruption of the tight junctions. Further *in vitro* studies are needed to address this and to underpin the link between IL-22, uterine endometrium regeneration, and inflammation-mediated infertility.

## Conclusion

IL-22 has pivotal importance in the prevention of secondary infertility with inflammation and in post-inflammatory regeneration by modulating tight and adherens junctions, orchestrating ECM proteins, and providing an antibacterial barrier. We demonstrated that IL-22 is a potential candidate for the treatment of secondary infertility and impaired uterine regeneration due to infection and inflammation that occur during the primary pregnancy.

## Data availability statement

The raw data supporting the conclusions of this article will be made available by the authors, without undue reservation.

## Ethics statement

This study was reviewed and approved by Animal Experimental Committee of Rosalind Franklin University of Medicine and Science.

## Author contributions

UG and SD developed the concept, interpreted the data, and wrote the manuscript text. UG, SS, and PB worked on data collection and analysis. UG prepared the figures. KB provided critical revision and editing. All authors contributed to the article and approved the submitted version.

## Funding

This work was supported by funds from the Clinical Immunology Lab, Center for Cancer Cell Biology, Immunology, and Infection at Rosalind Franklin University of Medicine and Science.

## Acknowledgments

We would like to express our immense gratitude to Patricia A. Loomis, PhD (Research Assistant Professor Department of Biomedical Research, Director: Microscopy and Imaging Facility, Electron Microscopy Facility at Rosalind Franklin University of Medicine and Science) for her mentorship and technical support in obtaining the confocal data for this manuscript. We are grateful to Alice Gilman-Sachs, PhD, for proofreading our manuscript and giving valuable comments.

## Conflict of interest

The authors declare that the research was conducted in the absence of any commercial or financial relationships that could be construed as a potential conflict of interest.

## Publisher’s note

All claims expressed in this article are solely those of the authors and do not necessarily represent those of their affiliated organizations, or those of the publisher, the editors and the reviewers. Any product that may be evaluated in this article, or claim that may be made by its manufacturer, is not guaranteed or endorsed by the publisher.

## Supplementary material

Supplementary Figure 1 IL-22^-/-^ mouse that managed to get pregnant after previous LPS-induced abortion. IL-22^-/-^ mouse had smaller implantation sites (indicated by black arrow heads) (~0.4 cm), in comparison to WT mice (~0.8cm). Harvested on gestational day 8.5. WT, wild type, IL-22^-/-^, IL-22 knockout.

Supplementary Figure 2 The expression of proteolytic enzymes in IL-22^-/-^ and WT mice. **(A)** IL-22^-/-^, n=6 and WT, n=5 mice uterine samples were harvested 48 hours post intraperitoneal LPS injection. Total mRNA samples were separated and processed by qRT-PCR to evaluate the expression of proteolytic enzymes (matrix metalloproteinases). The Ct values were normalized against the Ct values for GAPDH from the same preparation. The data are provided as mean ± SEM from 3 independent experiments. **(B)** IL-22^-/-^, n=6 and WT, n=5 mice uterine samples were harvested 48 hours post intraperitoneal LPS injection, frozen fixed, and processed for IHC analysis. H-score of MMPs was calculated to measure whether the difference of the staining between the groups is statistically significant. The data from t-test are provided as mean ± SEM. WT, wild type, IL-22^-/-^, IL-22 knockout, matrix metalloproteinase, MMP, H-score, histological score. Original magnification 40x, scale bar 100 µm.

Supplementary Figure 3 The expression of cytokeratin-8 in IL-22^-/-^ and WT mice. **(A)** IL-22^-/-^, n=6 and WT, n=5 mice uterine samples were harvested 48 hours post intraperitoneal LPS injection. Total mRNA samples were separated and processed by qRT-PCR to evaluate the expression of cytokeratin-8. The Ct values were normalized against the Ct values for GAPDH from the same preparation. The data are provided as mean ± SEM from 3 independent experiments. **(B)** IL-22^-/-^, n=6 and WT, n=5 mice uterine samples (half of one horn) were harvested 48 hours post intraperitoneal LPS injection, frozen fixed, and processed for IHC analysis. **(C)** H-score of cytokeratin-8 was calculated to measure whether the difference of the staining between the groups is statistically significant. The data from t-test are provided as mean ± SEM. WT, wild type, IL-22^-/-^, IL-22 knockout, H-score, histological score. Original magnification 40x, scale bar 100 µm.

Supplementary Figure 4 The expression of claudin-3 in IL-22^-/-^ and WT mice. **(A)** IL22^-/-^, n=6 and WT, n=5 mice uterine samples were harvested 48 hours post intraperitoneal LPS injection. Total mRNA samples were separated and processed by qRT-PCR to evaluate the expression of claudin-3. The Ct values were normalized against the Ct values for GAPDH from the same preparation. The data are provided as mean ± SEM from 3 independent experiments. **(B)** IL-22^-/-^, n=6 and WT, n=5 mice uterine samples (half of one horn) were harvested 48 hours post intraperitoneal LPS injection, frozen fixed, and processed for IHC analysis. **(C)** H-score of claudin-3 was calculated to measure whether the difference of the staining between the groups is statistically significant. The data from t-test are provided as mean ± SEM. WT, wild type, IL-22^-/-^, IL-22 knock out, H-score, histological score. Original magnification 40x, scale bar 100 µm.

Supplementary Figure 5 Immunofluorescence analysis of claudins and mucin-1. **(A)** The signals of claudin-2, claudin-10 and mucin-1 staining were stronger in WT mice in comparison to IL-22^-/-^ mice. **(B, C)** ROI lines were drawn to analyze fluorescence signals (blue for DAPI, green for cytokeratin) and to measure the height of the epithelial layer at individual cell level. **(D, E)** The histograms show the ROI lines for selected cells **(D** 1,2,3 in IL-22^-/-^ sample, **(E)** 1,2 in WT sample). Y axis is fluorescence intensity, X axis is length in µm. Original magnification 60x5, scale bar 10 µm. WT wild type, IL-22^-/-^, IL-22 knockout, ROI, region of interest.

## Abbreviations

ECM, extracellular matrix; H-score, histological score; IF, immunofluorescence; IHC, immunohistochemistry; IL-TIF, IL-10-related T cell-derived inducible factor; IL-22, interleukin 22; IL-22^−/−^, IL-22 knockout; gd, gestational day; LPS, lipopolysaccharide; MET, mesenchymal–epithelial transition; PCOS, polycystic ovary syndrome; RIF, recurrent implantation failure; RPL, recurrent pregnancy loss; RT-qPCR, reverse transcription quantitative real-time PCR; WT, wild type.
