# Supplementary figures and images for: IL-22 regulates endometrial regeneration by enhancing tight junctions and orchestrating extracellular matrix
Source: Front Immunol. 2022 Aug 25;13:955576. doi: 10.3389/fimmu.2022.955576 (PMC9453595; doi:10.3389/fimmu.2022.955576)

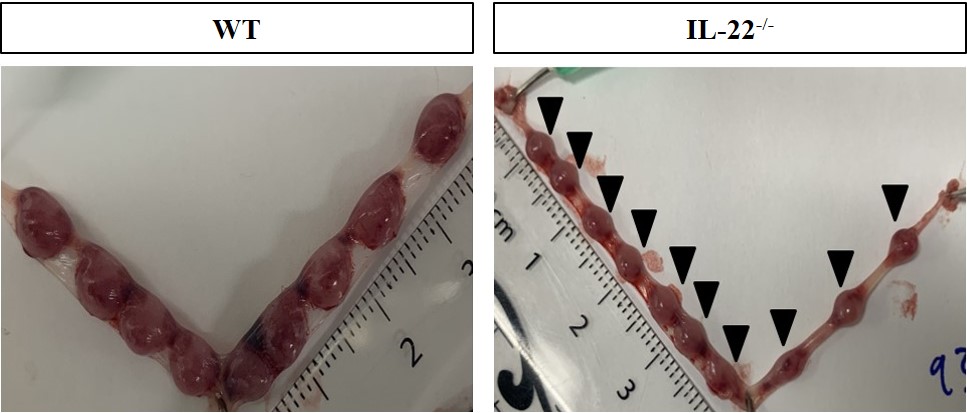

Supplement: Supplementary Figure 1 — IL-22-/- mouse that managed to get pregnant after previous LPS-induced abortion. IL-22-/- mouse had smaller implantation sites (indicated by black arrow heads) (~0.4 cm), in comparison to WT mice (~0.8cm). Harvested on gestational day 8.5. WT, wild type, IL-22-/-, IL-22 knockout. [file Image_1.jpg]

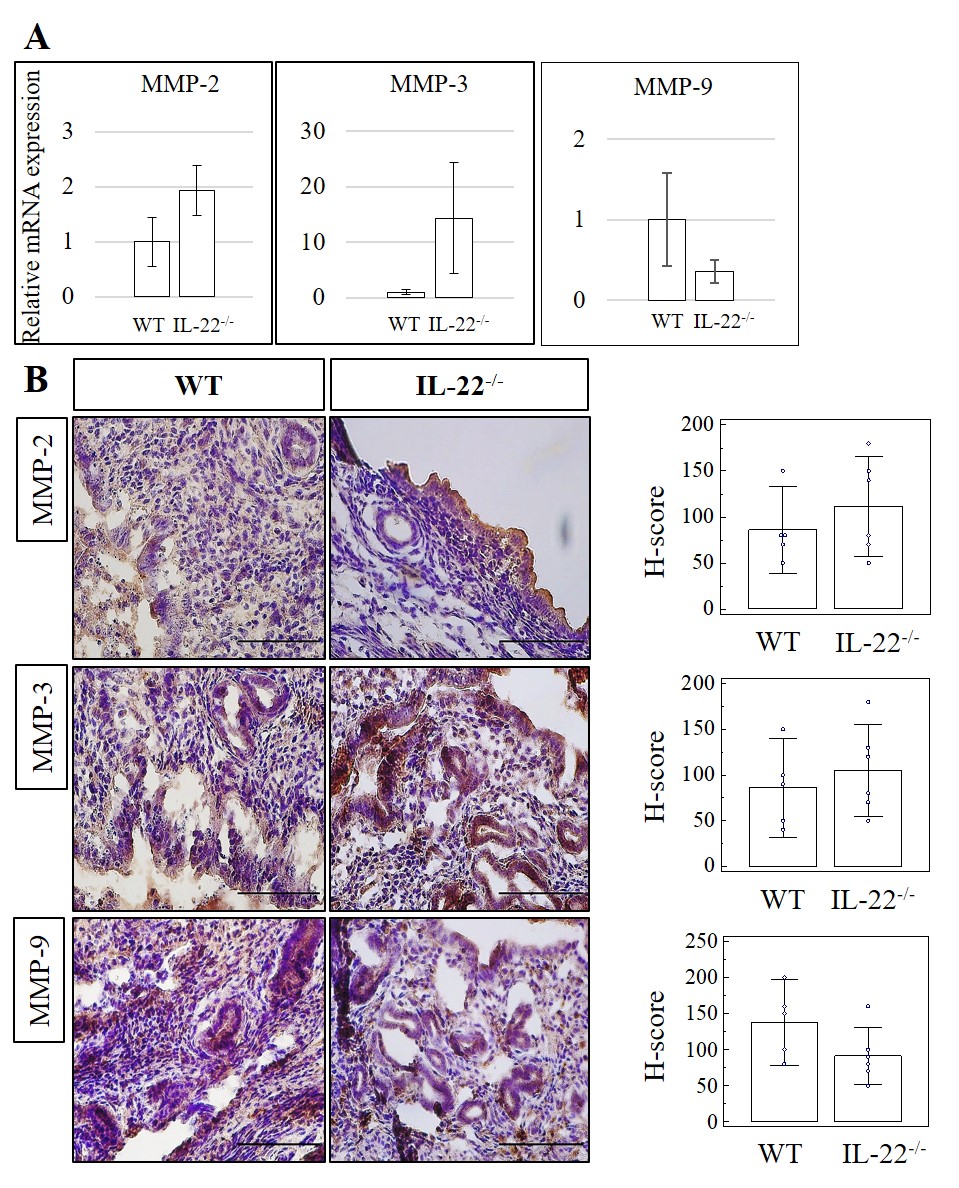

Supplement: Supplementary Figure 2 — The expression of proteolytic enzymes in IL-22-/- and WT mice. (A) IL-22-/-, n=6 and WT, n=5 mice uterine samples were harvested 48 hours post intraperitoneal LPS injection. Total mRNA samples were separated and processed by qRT-PCR to evaluate the expression of proteolytic enzymes (matrix metalloproteinases). The Ct values were normalized against the Ct values for GAPDH from the same preparation. The data are provided as mean ± SEM from 3 independent experiments. (B) IL-22-/-, n=6 and WT, n=5 mice uterine samples were harvested 48 hours post intraperitoneal LPS injection, frozen fixed, and processed for IHC analysis. H-score of MMPs was calculated to measure whether the difference of the staining between the groups is statistically significant. The data from t-test are provided as mean ± SEM. WT, wild type, IL-22-/-, IL-22 knockout, matrix metalloproteinase, MMP, H-score, histological score. Original magnification 40x, scale bar 100 µm. [file Image_2.jpg]

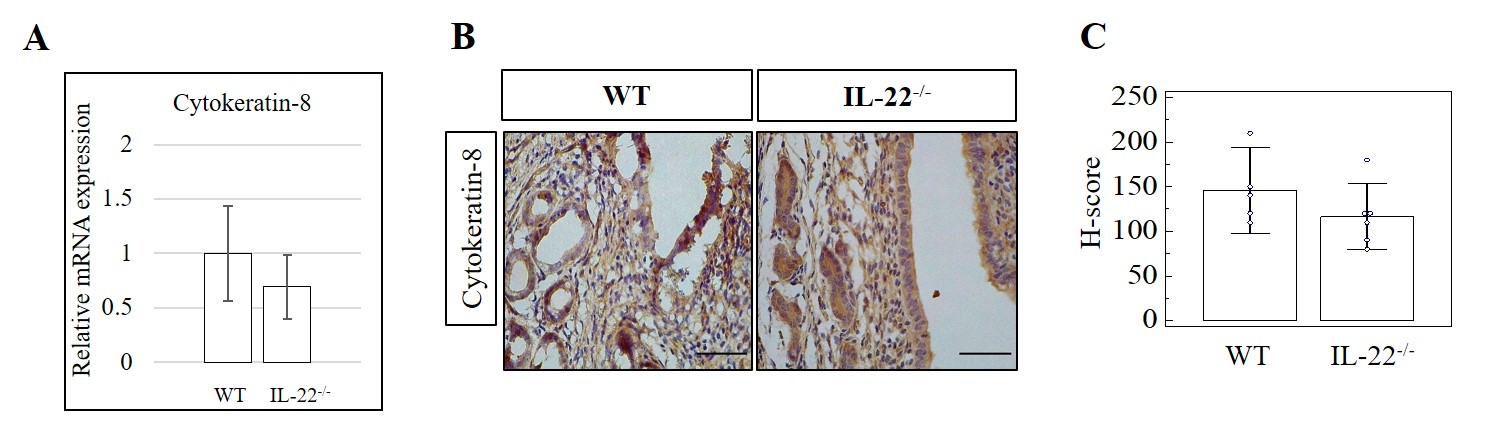

Supplement: Supplementary Figure 3 — The expression of cytokeratin-8 in IL-22-/- and WT mice. (A) IL-22-/-, n=6 and WT, n=5 mice uterine samples were harvested 48 hours post intraperitoneal LPS injection. Total mRNA samples were separated and processed by qRT-PCR to evaluate the expression of cytokeratin-8. The Ct values were normalized against the Ct values for GAPDH from the same preparation. The data are provided as mean ± SEM from 3 independent experiments. (B) IL-22-/-, n=6 and WT, n=5 mice uterine samples (half of one horn) were harvested 48 hours post intraperitoneal LPS injection, frozen fixed, and processed for IHC analysis. (C) H-score of cytokeratin-8 was calculated to measure whether the difference of the staining between the groups is statistically significant. The data from t-test are provided as mean ± SEM. WT, wild type, IL-22-/-, IL-22 knockout, H-score, histological score. Original magnification 40x, scale bar 100 µm. [file Image_3.jpg]

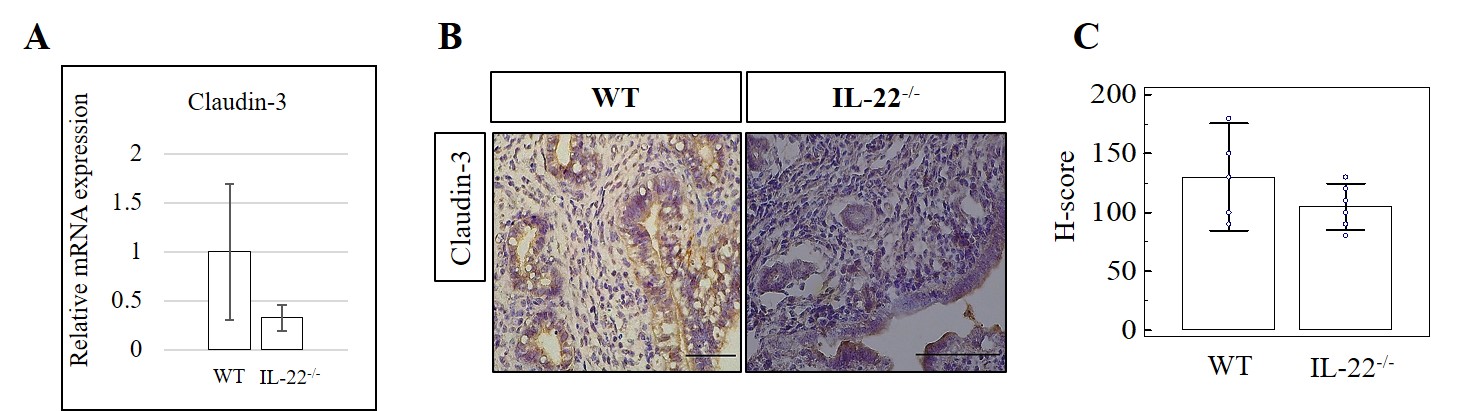

Supplement: Supplementary Figure 4 — The expression of claudin-3 in IL-22-/- and WT mice. (A) IL22-/-, n=6 and WT, n=5 mice uterine samples were harvested 48 hours post intraperitoneal LPS injection. Total mRNA samples were separated and processed by qRT-PCR to evaluate the expression of claudin-3. The Ct values were normalized against the Ct values for GAPDH from the same preparation. The data are provided as mean ± SEM from 3 independent experiments. (B) IL-22-/-, n=6 and WT, n=5 mice uterine samples (half of one horn) were harvested 48 hours post intraperitoneal LPS injection, frozen fixed, and processed for IHC analysis. (C) H-score of claudin-3 was calculated to measure whether the difference of the staining between the groups is statistically significant. The data from t-test are provided as mean ± SEM. WT, wild type, IL-22-/-, IL-22 knock out, H-score, histological score. Original magnification 40x, scale bar 100 µm. [file Image_4.jpg]

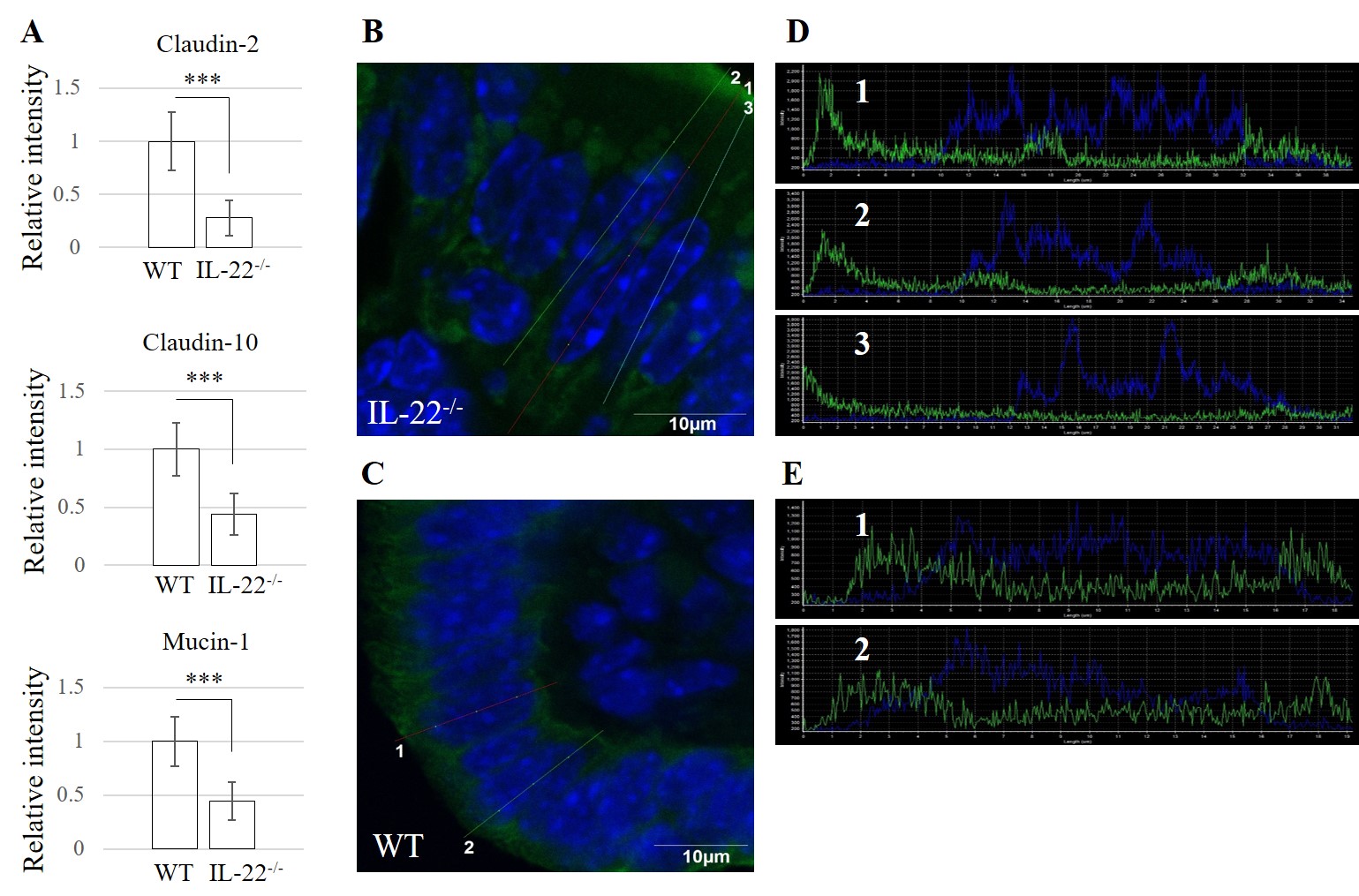

Supplement: Supplementary Figure 5 — Immunofluorescence analysis of claudins and mucin-1. (A) The signals of claudin-2, claudin-10 and mucin-1 staining were stronger in WT mice in comparison to IL-22-/- mice. (B, C) ROI lines were drawn to analyze fluorescence signals (blue for DAPI, green for cytokeratin) and to measure the height of the epithelial layer at individual cell level. (D, E) The histograms show the ROI lines for selected cells (D 1,2,3 in IL-22-/- sample, (E) 1,2 in WT sample). Y axis is fluorescence intensity, X axis is length in µm. Original magnification 60x5, scale bar 10 µm. WT wild type, IL-22-/-, IL-22 knockout, ROI, region of interest. [file Image_5.jpg]
